# More of less: Novel multi-ome profiling of single human neurons

**DOI:** 10.1016/j.xgen.2022.100110

**Published:** 2022-03-09

**Authors:** Huiwen Che, Bernard Thienpont

**Affiliations:** 1 Laboratory for Functional Epigenetics, Department of Human Genetics, KU Leuven, Leuven 3000, Belgium; 2 Aligning Science Across Parkinson’s (ASAP) Collaborative Research Network, Chevy Chase, MD 20815, USA; 3 KU Leuven Institute for Single Cell Omics (LISCO), KU Leuven, Leuven 3000, Belgium

## Abstract

Epigenetic modifications to DNA and chromatin interact to influence gene expression and cellular phenotypes, but defining these omics layers in complex tissues is a daunting task. In this issue of *Cell Genomics*, Luo et al. describe a novel single-cell multi-omic method, simultaneously profiling transcriptome, DNA methylome, and chromatin accessibility, to shed light on human neurons.

## Main text

Epigenetic changes are key in driving cell lineage choices and stabilizing cell identities. Defining the epigenome of individual cell types in complex tissues is a task that is often impossible, given the paucity of methods to isolate or purify specific cell types. This is especially true when accurate genetic labeling is unfeasible, for example, in patient samples or in complex tissues such as the brain.^1^, ^2^, ^3^ The advent of single-cell multi-omic methods has, however, brought this task into the realm of possibility: multi-omic methods enable the joint phenotyping of every cell for both its transcriptome and epigenome. In doing so, they circumvent the need to isolate every single cell type for an epigenome analysis. Not surprisingly, these recent advances are being met with great enthusiasm: single-cell multimodal approaches were selected as Method of the Year by *Nature Methods* just two years ago.^4^

**Figure 1:**
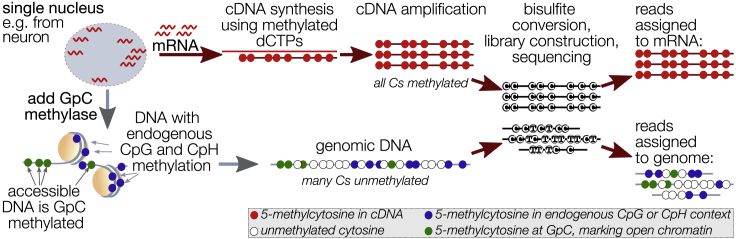
Schematic overview of the single-nucleus methylcytosine, chromatin accessibility, and transcriptome sequencing (snmCAT-seq) method, developed by Luo et al.^5^

In this issue of *Cell Genomics*, Luo et al. describe a novel method called single-nucleus methylcytosine, chromatin accessibility, and transcriptome sequencing (snmCAT-seq).^5^ Here, DNA in accessible chromatin is first labeled using a GpC methyltransferase. This open chromatin signature can then be detected by bisulfite sequencing, alongside the endogenous DNA methylation that predominantly occurs in a CpG context (Figure 1). The approach is akin to single-cell nucleosome, methylation, and transcription sequencing (scNMT-seq), which enables a joint analysis of these same omics modalities,^6^ but snmCAT-seq entails two key differences that set it apart from scNMT-seq. First, scNMT-seq requires a physical separation of DNA and mRNA to produce two separate sequencing libraries per cell, which adds cost, complexity, and hands-on time. However, Luo and colleagues came up with a neat trick to circumvent this need for physical separation: by replacing deoxycytidine triphosphate (dCTP) with 5-methyl-dCTP during complementary DNA (cDNA) synthesis, the resultant cDNA molecules carry a methyl at every cytosine, in contrast to genomic DNA.^5^ cDNA synthesis can then proceed directly on sorted nuclei, as reads from bisulfite sequencing can be readily assigned to either the transcriptome or genome through their cytosine content (Figure 1). The novel approach does have opportunity costs: the transcriptome cannot be sequenced separately (at a depth of choice) and fewer transcripts are detectable in each nucleus. Second, to amplify the genome, snmCAT-seq requires a single round of random priming, followed by enzymatic extension and adaptor annealing. scNMT-seq, however, involves several rounds of random priming before library preparation, which introduces significant bias and read chimerism. As a consequence, snmCAT-seq shows far better mappability than scNMT-seq, reducing the sequencing cost. scNMT-seq, however, seems to produce slightly more complex libraries than snmCAT-seq, covering a larger fraction of the genome. A direct head-to-head benchmarking is needed to better understand which method is preferable in a specific context. Currently, snmCAT-seq seems particularly advantageous for studies requiring more cells rather than more depth per cell.

Importantly, when applying snmCAT-seq to human neurons, Luo and colleagues describe a number of exciting opportunities and novel insights in both neurobiology and disciplines beyond. For example, a classical conundrum in single-cell analyses is figuring out if the parameters used to cluster cells by cell state or type are not overly permissive or restrictive, causing over- or underestimation of the number of cell types. Aiming at reaching a robust clustering, Luo and colleagues take advantage of the multimodal nature of their datasets, jointly optimizing cluster resolution in a co-training fashion.^5^ Specifically, they suggest that two sets of cells that are clustered as separate cell types in one modality should be considered a single cluster if, in another modality, cells between both sets are not more different than within that set. If the resolution offered by both omics modalities is similar, this indeed seems a worthwhile and promising assumption. Fine-grained assessment of cluster qualities using graph-based similarities is implemented by the authors to identify informative clusters and to infer cell sub-populations that are supported by multiple omics layers and are thus more likely to be biologically meaningful.

An exciting epigenetic insight of the study concerns the endogenous methylation of cytosines outside the canonical CpG dinucleotide context, i.e., in the so-called CpH context. While non-CpG methylation is rare in most cells, neurons (along with stem cells and muscle cells) were previously shown to harbor high levels of CpH methylation in their gene bodies, with levels rising particularly postnatally.^7^ A recent study also highlighted that presence of CpH methylation in the brain is conserved among vertebrates, which suggests functional importance.^8^ Nevertheless, the function of CpH methylation remains unclear. By comparing CpH methylation with gene expression in single cells, Luo and colleagues, however, observe a number of peculiar correlations. For example, 38% of gene bodies showed an inverse correlation between CpH methylation and gene expression, in line with earlier observations,^7^ but the remainder of genes showed no correlation, and some even showed substantial variation in CpH methylation between cell types without corresponding variation in RNA expression. Luo et al. show that genes without correlation between CpH methylation and expression are often downregulated during neuronal development and marked by H3K27me3, suggesting that different subsets of genes are under divergent epigenetic control. While functional consequences of CpH methylation remain to be established, these observations already provide an in-route in which genes and regulatory pathways are to be studied.

Given the need to better understand the interplay between the epigenome and transcription in complex tissues, it is very encouraging to see that studies such as Luo et al.^5^ continue to push the envelope of single-cell technologies. Nevertheless, room and demand for further improvement remains. For example, a downside of bisulfite-based methods is their inability to distinguish 5-methylcytosine from 5-hydroxymethylcytosine. As this DNA demethylation intermediate is highly abundant in the brain,^7^^,^^9^ methods specific for either base will likely provide an even finer-grained picture of the DNA methylation landscape and its dynamics. Furthermore, the development of cost-effective droplet-based microfluidic methods has proven a strong booster for single-cell transcriptome and open chromatin studies of the brain in a number of organisms.^2^^,^^3^^,^^10^ Analogous development of multimodal methods that enable high-throughput probing of DNA methylomes at a lower cost will likely produce a further step change in the field of epigenetics.
